# Functional Validity of a Judgment Skills Measure within the Concept of Health Literacy for Sleeping Disorder Patients

**DOI:** 10.3390/ijerph111010868

**Published:** 2014-10-17

**Authors:** Arthur Dubowicz, Peter J. Schulz

**Affiliations:** Institute of Communication and Health, University of Lugano, Via G. Buffi 13, 6904 Lugano, Switzerland; E-Mail: peter.schulz@usi.ch

**Keywords:** health literacy, judgment skills, health behavior, patient decision, sleeping disorders

## Abstract

The concept of health literacy has been widened to include higher order aspects such as patient decision-making skills while its measurement continued to rely narrowly on reading and numeracy skills, known as functional health literacy. We developed a Judgment Skills measure, designed to assess patients’ ability to make appropriate decisions with regard to their condition. The measure offers scenarios with answer options ranked for biomedical adequacy. This study aims to examine the psychometric properties and the functional validity of the Judgment Skills measure. A self-administered survey among 87 primary insomnia patients in the Italian-speaking part of Switzerland was conducted. The extensive path model included variables such as functional health literacy, coping with the medical condition, experience of the scenario, sleep quality, duration suffering, education, and age. Correlation analyses were conducted to link the variables. The Judgment Skills measure showed the expected significant correlations. In general, higher Judgment Skills were related to coping strategies leading to better health outcomes. Functional health literacy correlated highly with education, while Judgment Skills did not, which confirmed the conceptual difference of these skills. The findings propose a model for conducting research that does embrace the broader conceptualization of health literacy.

## 1. Introduction

The concept of health literacy is built on general literacy, [1] and refers to reading and numeracy skills of health care consumers and their ability to make appropriate health-related decisions [2]. Conceptually health literacy is defined as the degree to which individuals have the capacity to obtain, process, and understand basic health information and services needed to make appropriate health decisions [3]. Many studies focus on health care consumers’ understanding of providers, treatment advice, or health information. Additionally, the concept of health literacy is also applied to measuring the comprehensibility of health information issued to consumers [4,5]. The concept of health on literacy also includes cultural factors and conceptual knowledge [6,7]. Multiple measures for health literacy are available, taking different domains and contexts into account [1].

When measuring health literacy, most instruments focus on the functional components, that is reading and numeracy skills. The widely used and validated instruments for measuring health literacy such as the Test of Functional Health Literacy (TOFHLA), the Rapid Estimate of Adult Literacy in Medicine (REALM) and their short versions, and the Newest Vital Sign scale, are limited to the functional and knowledge components of health literacy [8,9,10]. Besides these instruments, review articles identified a wide quantity of different scales that can be used for assessing health literacy [11,12]. Despite the huge number of scales, none of the tools captures the conceptual definition of health literacy entirely [13,14]. Most research focuses on the functional aspect of health literacy, while the other aspects are addressed only sparsely [14]. Research indicates that this narrow focus must be overcome by providing instruments for measuring the higher order aspects of health literacy, meaning measures that provide information on how medical information is processed and used by health care consumers [11,14].

To address these restrictions, Schulz and Nakamoto proposed a patient-centered model of health literacy focusing on Judgment Skills, [15,16,17] while retaining the components of functional health literacy: declarative knowledge and procedural knowledge [17]. The advantage of including a scale for Judgment Skills allows taking factors relating to knowledge, personal competence, and general practical intelligence into consideration when an independent patient decision for behaving in a certain way is under scrutiny. Judgment Skills is defined as the ability of patients to make sound autonomous medical decisions and to manage their condition on the basis of distinct knowledge. The conceptualization of Judgment Skills as patient decisions takes the impact of these skills on the personal health outcome into consideration and is linked to individual cognitive and behavioral capabilities. This means that patients make their decisions based on previous knowledge, personal experiences from past medical encounters, and coping strategies developed then. The decision is situation-specific and influenced by factors that relate to empowerment and components of health literacy other than Judgment Skills [18,19]. Based on the Schulz and Nakamoto model, we developed a measure of Judgment Skills in persons with sleeping disorders, as reported elsewhere [20]. The measure presents scenarios and asks respondents which of four behavioral responses s/he would choose in a given situation.

The present study aims to examine the psychometric properties and the functional validity of the Judgment Skills measure with a sample of Swiss participants suffering from sleeping disorders. A comprehensive path model incorporating several research-driven assumptions was developed. Additionally to the Judgment Skills measure, a scale of functional health literacy was included. A coping scale was used to describe patients’ efforts in mastering their condition. This measure does not refer to traditional coping scales but is based on prominent issues mentioned by sleeping disorder patients in previous focus groups. The correlations of all pathways were grouped according to their relevance for the Judgment Skills measure. Paths directly related to Judgment Skills were seen as crucial for the validation process. In detail, it is considered in the model that participants with high Judgment Skills are likely to adopt medically sound decisions and have improved sleep quality. In addition, a correlation with coping is expected as this can be found in the literature [21] (Paths b & d). To assess the relevance of the measure, participants were asked to indicate if they had experienced situations similar to the scenarios. Research has shown that experience with a medical condition is linked to better management [22]. Based on the assumption that both measures of functional health literacy and the Judgment Skills scale measure aspects of health literacy, we expect the two scales to be correlated (Path e).

Further paths were grouped as ancillary for the validation argument. Research has shown that the duration of suffering from a disease influences patient behavior, therefore correlations are included between the duration and Judgment Skills, either directly or via experience, and between Judgment Skills and the outcome of sleep quality, either directly or by coping (Paths a, c, f, g) [23].

**Figure 1:**
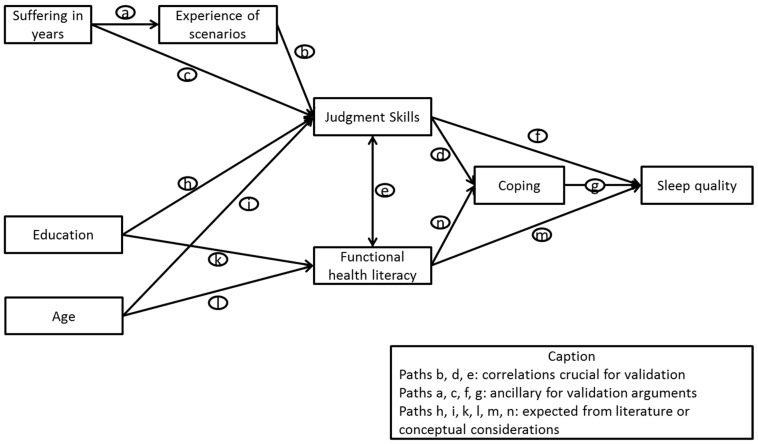
Expected correlations in the validation path model.

Another set of paths is either extrapolated from literature or based on conceptual considerations and informed experience from the pretest (Paths m & n). In contrast to functional health literacy, no correlations are expected to be found between education and age on the one side and Judgment Skills on the other. These correlations were established in previous studies for the functional component (Paths h, I, k, l) [21,24,25]. Regarding age, cognitive capabilities tend to decrease while experience with certain situations increases, leading to different influences on Judgment Skills balancing each other out. Education and functional health literacy rely both on literacy, while Judgment Skills is based on cognitive and social skills. Conceptually, correlations between functional health literacy with coping and the outcome measure are expected. The path model is shown in Figure 1. By assessing the psychometric properties of the Judgment Skills measure, this study can provide findings on components of the measure which need further adapting or to be redefined.

To measure Judgment Skills we chose the domain of sleeping disorders. This medical condition constitutes one of the most common complaints in primary care and is highly prevalent in the study area, with 39.8% of the population reporting sleeping disorders [26,27,28,29]. Research has identified adherence to the medical regimen as challenging for sleeping disorder patients [30]. Medication misuse is well known as a major problem with significant side-effects. Benzodiazipine is widely used and prone to side-effects and misuse in the treatment of sleep disorders [31,32]. For measuring the occurrence of such patient decisions simulated scenarios regarding medication use and adherence were incorporated into the Judgment Skills measure (Table 1). Sleeping disorder patients often have to manage side-effects autonomously, without decreasing the efficacy of the sleep medication. Moreover cognitive behavioral therapy requires them to maintain complex sleep diaries on their own [33]. Literature acknowledges that the management sleeping disorders requires high health literacy as medical outcome is based on patient participation [33], but not much research on this relationship can be found. In contrast to medical conditions such as cancer, sleeping disorders have a less significant emotional and psychological component and bear only little controversy regarding treatment.

**Table 1:** Items of the Judgment Skills measure.

Medication use and adherence	
1	Your doctor prescribed you a medicine for sleeping disorders. Even though you are sticking to the medical regimen, you can’t fall asleep. How would you react?
2	You are in bed for more than half an hour and can’t fall asleep. The next day you have an important meeting with your boss/an important family event. What would you do?
3	A friend tells you about the positive effect of a certain medication, but your doctor doesn’t want to give you a prescription. What would you do?
4	Your doctor has prescribed you a strong medication for sleeping disorders. You should use this medication only when your usual medication isn’t helping, and you’ve had more than three nights with complaints. You couldn’t sleep last night, and tomorrow you have an important meeting. What would you do?
5	A friend or family member of yours has been having problems falling asleep for several nights and has been staying awake during the day. Your friend is desperate and asks you for prescription medication to get some sleep, and finally feel better. What would you do?
6	At the moment you have difficulties falling asleep. A friend or family member currently takes a medication for sleeping disorders that works well. Since you have sleeping problems yourself these days, what would you do?
**Self-management and knowledge**	
7	Some friends tell you about a new medication for sleeping disorders and their positive experience with it. How would you react?
8	In the media you heard of a new alternative medication and some home remedies for sleeping disorders. What would you do?
9	You’ve been having sleeping problems, so you went to your doctor. The doctor asked you about your personal situation, your workplace setting, and your general medical condition. The doctor talks to you about managing sleeping disorders without a medication and doesn’t give you a prescription. How would you react?
10	For a while you have been taking a medication for sleeping disorders that contains benzodiazepine. These days this medication isn’t working very well for you. The doctor told you to stick to your medical regimen, but you know that slightly overdosing on benzodiazepines isn’t dangerous. What would you do?
**Social recognition**	
11	You realize that your sleeping disorders disturb your bed partner’s sleep. What would you do?
12	You stop by a pharmacy for some headache medicine. You realize that you also need a refill of your prescribed medication for sleeping disorders, but you left your prescription at home. The pharmacist says that you can’t get the medication without your prescription in hand. How would you react?
13	During the day it’s difficult for you to stay awake. You suffer from daytime fatigue and sleepiness. But as you have an appointment in the evening, you want to look refreshed. What would you do?
14	You are planning a weekend trip with friends. As there are a limited number of rooms in the hotel, you have to share the room with someone else. What would you do in order to avoid your roommate being disturbed by your sleeping problems?
**Consumption of sleep-active substances**	
15	For several nights you can’t sleep properly. What would you do?
16	Your medication works best when you reduce the consumption of caffeine, tobacco, and alcohol. You are at a party where everyone is drinking alcohol, and you are offered an alcoholic drink. What would you do?

## 2. Methods

### 2.1. Participants

All participants of this study had a Swiss health-care plan and were recruited through the sleep-center at the cantonal hospital in Lugano, Switzerland (Ospedale Regionale di Lugano—Civico, Neurocentro della Svizzera Italiana, Cento del sonno) or through general practitioners in the city. They had been diagnosed with primary insomnia and were receiving cognitive behavioral therapy [34]. A self-administered questionnaire was distributed to patients who agreed to participate; it was filled out while patients were in the hospital or the doctor’s office. Inclusion criteria were a minimum age of 18 years and suffering from sleeping disorders for at least one month.

The research protocol of this study was approved by the Ethical Committee of the responsible governmental health authority and the Research Committee of the collaborating hospital. Participants received a consent form, as approved by the Ethical Committee. As they were handed the questionnaire, participants received oral and written information about the study. During the time patients filled out the questionnaire, research staff was available for questions. When returning the questionnaire, participants received a 10 CHF voucher for a department store.

### 2.2. Measures

#### 2.2.1. Judgment Skills

Sixteen scenarios with four answer options each were presented (Table 1). The scenarios describe typical everyday challenges, which patients may encounter. The answer options provide different behaviors in reaction to the scenario situation; the patient was to pick one. The process of scale construction involved experts in the field who commented and reviewed the items over several rounds, as reported elsewhere [20]. Based on the assessment of the biomedical adequacy of each option by the medical experts, the four options were ranked from 1 = best to 4 = worst. To get a single measure, the ranks were averaged up, per respondent and the resulting scores then dichotomized along the median (1.7). A lower mean indicates higher Judgment Skills by the participant. The Judgment Skills items cover the four dimensions of medication use and adherence (6 scenarios), self-management and knowledge (4 scenarios), social recognition (4 scenarios), and lifestyle factors such as consumption of sleep-active substances (2 scenarios). Five versions with randomized questions were handed to the participants to avoid ordering effects.

#### 2.2.2. Experience of Scenario

Participants were asked on a five point scale how often they had experienced the situation described in the scenario. In this way, the relevance of the presented scenario for the patients was investigated. This research layout also allowed differentiating patients who had experienced a scenario and those who had not. For each participant a mean score based on the sum of all 16 items was calculated and used for analysis. These items were also used to confirm the practical significance of the situations presented in the Judgment Skills measure.

#### 2.2.3. Functional Health Literacy

A measure of health literacy had to be found that would work within a self-administered questionnaire. We chose the three item version of the existing measure [21], which uses situations patients typically encounter in a medical setting. In its development process the items were checked against the short form of the TOFHLA [8] and revealed to be reliable predictors for patients’ functional health literacy level [21,24]. In data analysis, a mean score for functional health literacy was computed for each participant, who was then assigned to one of the three groups: inadequate, marginal, adequate health literacy. For this research a three item Italian version of the measures was used. Previous research showed that this measure can be applied within this study population in the context of the Swiss health care system [25].

#### 2.2.4. Epworth Sleepiness Scale

This scale provides a measurement of the participants’ level of daytime sleepiness and allows estimating the general severity of the sleeping disorder. The measure consists of eight items, which describe situations sleeping-disorder patients typically encounter [35]. This measure has proven to be reliable in providing satisfactory results. For this research the Italian version of the scale was used [36]. For each participant a sum score was calculated and they were grouped accordingly into one of three groups, as proposed by the authors of the measure. The groups were labeled as dangerous, problematic, and normal. This measure was used to ensure a balanced sample of sleeping disorder patients for this study. The scale was included to allow comparing the study population and the cantonal population affected by sleeping disorders [29].

#### 2.2.5. Coping with the Condition

This is an eight item scale, composed of personal statements, which were collected in a focus group with sleeping disorder patients in previous research and pre-tested together with the Judgment Skills measure [20]. The measure describes how patients are mastering their medical condition with regard to a better health outcome. Participants were asked to share their opinion on a five point scale from disagree to agree. The statements are oriented on the four groups of the Judgment Skills measure: medication use, self-management, social recognition, and consumption of sleep active substances. This scale provides an estimate of participants’ self-assessment of their abilities to treatment adherence. Section 3.3. provides an overview of the eight items.

#### 2.2.6. Additional Variables

On the last page of the questionnaire participants were asked socio-demographic characteristics. They included gender, year of birth, educational background, and their postal code, to ensure residence within the area of research, and to be certain they had comparable health care coverage. The participants were asked how their general sleep quality was in the past seven days, and were asked to rank it on a four point scale. They were also questioned regarding the duration of suffering from sleeping disorders and employment in the medical sector.

### 2.3. Data Analysis

The whole dataset was checked for missing values. In all scales (Judgment Skills, personal relevance, functional health literacy, Epworth sleepiness scale, coping with the condition) missing values were replaced if less than equal to 5% missing values occurred in one scale. Following this rule three cases were excluded from further analysis. One item (11) of the Judgment Skills scale was excluded from the data analysis, as it contained a misleading assumption (participants have a bed partner). Thus the Judgment Skills scale contained 15 items for analysis. During the data analysis we controlled for age and education. The scale correlations (Pearson) and differences in coping were calculated using group comparisons and scale reliability analysis. The analysis followed the structure of the extensive theoretical path model but is not to be mistaken with a path analysis. The data was analyzed using IBM SPSS version 21 [37].

## 3. Results

### 3.1. Socio-Demographic Characteristics

Of the 87 participants 54 (62.1%) were female, 31 (35.6%) male and two (2.3%) didn’t provide an answer to this question. The age range was 25 to 73 years resulting in a mean age of 50.61 years (SD 10.82). The gender and age characteristics found in this study are typical for sleeping disorder patients [38]. Participants had been suffering from sleep disorders for 10.95 years on average (SD 10.82). The majority of participants had finished high school and a form of secondary education or university degree; the numbers roughly match the Swiss population’s educational level [39]. Four participants (4.6%) reported to work in the medical sector. Participants needed about 25 minutes to fill out the entire questionnaire. Findings of the Epworth Sleepiness scale were discussed with a sleep expert to ensure that the participants of the study and patients on a cantonal level were comparably affected by sleeping disorders. Summarized details of the participants’ characteristics can be found in Table 2.

**Table 2:** Participants of the study.

Variable	N	%
All participants	87	100
*Gender*		
Male	31	35.6
Female	54	62.1
Missing	2	2.3
*Age group*		
25–39	16	18.4
40–49	22	25.3
50–59	31	35.6
60–73	15	17.2
Missing	3	3.4
Mean 50.61 years; SD 10.82		
*Education*		
No obligatory school	1	1.1
Obligatory school	6	6.9
Apprenticeship	33	37.9
High school	16	18.4
Professional school	13	14.9
University	16	18.4
Missing	2	2.3
*Suffering from sleeping disorders*		
Mean 10.95 years; SD 7.83		
*Epworth Sleepiness Scale*		
Dangerous situation	50	57.5
Problematic situation	13	14.9
Normal situation	24	27.6
*Functional health literacy*		
Inadequate	1	1.1
Marginal	54	62.1
Adequate	32	36.8
*Judgment Skills*		
Low	46	52.9
High	41	47.1

### 3.2. Reliability and Construct Validity

The internal consistency of the 15 item Judgment Skills scale was adequately high (Cronbach α 0.853). The inter-item correlations were all positive, with two exceptions (item 2 with 12: −0.016 and 3 with 16: −0.006), ranging from −0.016 to 0.545. All remaining 15 items were kept, as deleting items would not lead to higher scale reliability. As in previous work on developing the Judgment Skills scale, the items comply with the test specification [20].

### 3.3. Differences in Coping

In general, participants with higher Judgment Skills had fewer difficulties coping with their condition. Medication sharing or over-medication seems to be less of a problem (Item 1 & 2). When it came to the correct use of their medication, they were more confident about the doctor’s advice (Item 4), and had fewer problems following the medical regimen (Item 6), and had better control over the intake of sleep-active substances such as alcohol (Item 3). The group with a higher Judgment Skills score also reported more often to be well treated by their medical doctor than the group with a lower score (Item 5) and could improve their physical condition by following the medical treatment more easily than patients with a lower score (Item 7). Participants with high and low Judgment Skills reported daytime impairments due to their medical condition on an equally high level (Item 8). A summary of these findings can be seen in Table 3.

**Table 3:** Agreement to items of the coping measure (in %).

Item of the Coping Measure		Judgment Skills		Chi^2^	df	*p*
		High(n = 41)	Low(n = 46)			
		%	%			
1	I wouldn’t share my medication with others	80.5	47.8	9.95	1	0.001
2	I wouldn’t take more than my prescribed medication	70.7	56.6	1.87	1	0.13
3	I wouldn’t drink alcohol while the time I’m on medication	68.4	50.0	2.52	1	0.09
4	It is no problem for me to follow my doctor’s advice	65.9	47.8	2.85	1	0.18
5	I feel well treated by my doctor	65.9	41.3	5.23	1	0.02
6	It is no problem for me to follow my prescribed medication	51.5	36.6	1.66	1	0.15
7	I feel better when I follow my medical treatment	41.5	32.6	0.72	1	0.25
8	My disease doesn’t cause big daytime impairments for me	24.4	23.9	0.003	1	0.58

### 3.4. Scale Correlations in the Path Model

Judgment Skills prove to be significantly correlated with several other variables, for instance experience of the presented situation (r = −0.232, *p* < 0.05). This might indicate that a certain level of familiarity with the presented situation might lead to a better response to it. A highly significant correlation with the functional health literacy measure was found (r = −0.336, *p* < 0.01). Participants with a higher level of Judgment Skills tend to have a higher share of adequate functional health literacy level. Higher Judgment Skills also showed to be related to better coping with the medical condition (r = −0.505, *p* < 0.01). No direct, significant correlation with Judgment Skills was found For sleep quality and the duration of suffering from sleeping disorders, but duration of the condition was correlated with experience (r = −0.228, *p* < 0.05) and sleep quality with coping, so that both were indirectly linked with Judgment Skills. The socio-demographic variables, like gender, age, and education did not correlate significantly with Judgment Skills.

**Figure 2:**
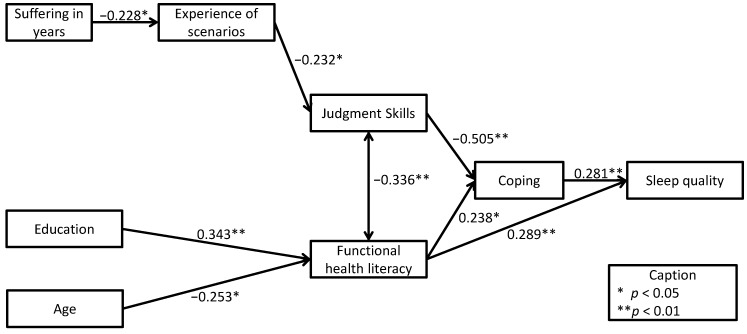
Correlations in the validation path model.

Experience with the presented situation was significantly related to the coping scale (r = 0.238, *p* < 0.05). Functional health literacy was related significantly to sleep quality (r = 0.289, *p* < 0.01), education (r = 343, *p* < 0.01), coping (r = 0.238, *p* < 0.05), and age (r = −0.253, *p* < 0.05). All correlations can be found in Table 4, and the important ones are illustrated in Figure 2.

**Table 4:** Pearson correlation matrix for the scales.

Scales	Judgment Skills	Experience of Scenarios	Functional Health Literacy	Coping	Sleep Quality	Suffering in Years	Education
Experience of scenarios	−0.232 *						
Functional health literacy	−0.336 **	0.083					
Coping	−0.505 **	0.238 *	0.238 *				
Sleep quality	−0.159	0.098	0.289 **	0.281 **			
Suffering in years	0.055	−0.228 *	−0.012	0.128	0.017		
Education	−0.141	−0.089	0.343 **	0.116	0.045	0.154	
Age	0.004	0.074	−0.253 *	0.074	−0.150	0.148	−0.120

Notes: * *p* < 0.05 (2-tailed); ** *p* < 0.01 (2-tailed); Pearson correlation coefficients (*N = 87*).

## 4. Discussion and Conclusions

### 4.1. Discussion

The measure for Judgment Skills is an attempt to investigate the broader concept of health literacy, seen as the ability to obtain, process, and understand health information and act appropriately within a health-care setting [1,3]. The notion of Judgment Skills offers the possibility to enrich the catalogue of existing (functional) health literacy measures, by providing a scale for the patient’s capacity of making biomedically appropriate decisions to manage their health condition. The relationships of the Judgment Skills measure and the other constructs of the path model were generally as hypothesized. Especially with regard to functional health literacy, the researches assumptions were confirmed and supported the construct validity of the measure. All crucial paths around the Judgment Skills items loaded significantly (Figure 2). The data showed that higher Judgment Skills were associated with more effective coping and would lead to better sleep quality. In general, patients who suffer longer periods of time had more experience with the situations presented in the Judgment Skills measure confirming the literature-driven hypothesis of a link between experience with medical situations and better management [22]. The findings indicate that sleeping disorder patients, regardless of their Judgment Skills level, report a high level of daytime impairment. However, especially participants with higher Judgment Skills seem to be more responsible when dealing with their sleep-medication and tend not to share medication with others. These patients have a stronger subjective feeling of being well treated by the medical staff. Research focusing on functional health literacy has indicated that low health literacy is related to communication problems in the medical sector, which might give an explanation for this finding [5,40].

Judgment Skills does not correlate with education in this study, but with coping. This finding proves the assumption that this measure is not related to formal education, but covers a set of skills linked to the patient’s experience in dealing with the medical condition, in contrast to functional health literacy, which shows a direct link to education [41,42]. Judgment Skills is also not a proxy of the participant’s age as often found regarding functional health literacy [41]. This absent link goes in congruence with findings of another approach for a measure designed to capture communicative and critical health literacy skills of patients. This concept is trying to investigate the aspects of obtaining and processing of medical information and also did not show a correlation with age [43,44].

This study bears several limitations. The patient population suffers from the same medical condition, as a specially adapted measure was used. Comparisons with other conditions are therefore impossible with the data at hand. For further research a bigger sample of this population would be desirable. With the chosen form of recruitment a quite diverse group of participants could be reached in the study area, but the general education level is quite high, with more than 90% of the participants having at least an apprenticeship or higher education (Table 2). Research in the field suggested that socioeconomic factors such as education, age, and income might be potential confounders in investigating health literacy. A further limitation is the fact that this measure is based on a self-administered questionnaire. Some participants might have filled it out with external help or might have reported socially desirable results.

### 4.2. Conclusions

This research provides a scale which is able to measure patients’ Judgment Skills, with a tested instrument. The definitions of health literacy are based on a wider concept than most of the current tools cover. Especially in highly literate societies and countries, measures for functional health literacy struggle to find people with low general health literacy [41]. Therefore, a measure different from knowledge and education can help to capture these groups, as barriers in health communication and health disparities still exist in the medical settings of these societies. The Judgment Skills measure contributes to closing the gap between the definition of health literacy and the available measures. The design of this measure does not only capture aspects of the health-care environment, but also takes into consideration the necessary and health-relevant decisions of patients with sleeping disorders outside the doctor’s practice.

This measure can be used for further research in the field of health literacy and patients with sleeping disorders to improve health communication and doctor-patient interaction. In its development, health care workers and patients have contributed to this measure and helped to create a realistic and relevant scale which takes cultural particularities into consideration. With ongoing further data collection among this population, it might be possible to provide a shorter version of the Judgment Skills measure and more precisely defined cut off points for Judgment Skills levels. The findings that already exist are a step towards a more effective health communication tool.
